# Evaluating the use of diagnostic CT with flattening filter free beams for palliative radiotherapy: Dosimetric impact of scanner calibration variability

**DOI:** 10.1002/acm2.70040

**Published:** 2025-02-19

**Authors:** Madeleine L. Van de Kleut, Lesley A. Buckley, Elsayed S. M. Ali

**Affiliations:** ^1^ Department of Radiology, Radiation Oncology and Medical Physics The Ottawa Hospital Ottawa Canada

**Keywords:** CT‐RED curve, diagnostic CT, flattening filter free, Hounsfield unit, palliative radiotherapy, volumetric‐modulated arc therapy

## Abstract

**Purpose:**

Palliative radiotherapy comprises a significant portion of the radiation treatment workload. Volumetric‐modulated arc therapy (VMAT) improves dose conformity and, in conjunction with flattening filter free (FFF) delivery, can decrease treatment times, both of which are desirable in a population with a high probability of retreatment with large palliative doses per fraction. Combining FFF and VMAT delivery with planning based on previously acquired diagnostic computed tomography (CT) scans has the potential to further expedite palliative treatment. This study evaluated the dosimetric uncertainty of using FFF beams with VMAT delivery on CT images acquired from different diagnostic vendors, and between different x‐ray tube energies, in the palliative setting.

**Methods:**

CT‐relative electron density (CT‐RED) curves were acquired for the local CT simulator at 100, 120, and 140 kVp, and for two diagnostic CT scanners at 120 kVp. Thirty palliative VMAT plans were recalculated for each CT‐RED curve, with 6 MV flat, 6 FFF, and 10 FFF beams. The doses to 95% and 2% of the PTV, the maximum point dose to the spinal canal and esophagus, and the mean dose to the kidneys were compared between recalculated plans.

**Results:**

Comparing the dose clouds for a given fluence map calculated with CT‐RED curves from different CT scanners at 120 kVp, the mean dose difference was at most 0.3% for each DVH metric. Similar results were reported when comparing dose clouds calculated with CT‐RED curves for 100, 120, and 140 kVp on the CT simulator.

**Conclusion:**

The results of this study confirm that diagnostic scans acquired on machines different from the CT simulator associated with the TPS, are appropriate for VMAT treatment planning in the palliative setting with FFF photon beams.

## 1 INTRODUCTION

Radiotherapy planning and delivery techniques continue to evolve to provide maximal target control while minimizing dose to healthy tissue. The widespread adoption of conformal techniques is one such method of achieving an optimal dose distribution. Palliative radiotherapy, traditionally treating larger target volumes, has historically been planned with a simpler forward‐planning technique despite the availability of more conformal approaches, as placed fields allow for greater setup variability in patients where positioning reproducibility may be compromised due to pain.

Advances in systemic therapy, surgery, and radiotherapy have led to improved and lengthened quality of life for many patients, resulting in the potential for multiple palliative radiation retreatments. Investigations into the use of volumetric‐modulated arc therapy (VMAT) for palliative cases, both in expedited rapid access settings and traditional planning pathways have been conducted.^1^, ^2^, ^3^ These studies highlight the utility of VMAT in the palliative setting, demonstrating an improved therapeutic ratio by decreasing toxicity to surrounding organs at risk, and leaving dosimetric room for future palliative treatments that may be abutting or overlapping. Feasibility from a planning and delivery perspective was also evaluated. With the routine use of cone beam computed tomography (CT)‐guided setup, VMAT is appropriate in the palliative setting, allowing for dose escalation and reduced treatment times. More recently, the validation and use of commercial solutions for deep learning or atlas‐based autocontouring of organs at risk has also led to a decreased treatment planning workload, further promoting the routine use of conformal techniques.^4^, ^5^, ^6^

In addition to conformal therapy, the use of flattening filter free (FFF) delivery is increasing, as the high dose rate allows for further shortened treatment times, and modulated delivery allows for compensation of non‐uniform photon fluence.^7^, ^8^, ^9^ In palliative cases where large doses are delivered in single or few fractions, decreased treatment time is ideal for patient throughput, to reduce the impact of spontaneous intrafraction motion, or drift with time, and to reduce the patient inconvenience and discomfort associated with lying in the treatment position. Compared to a flattened beam of the same nominal energy, FFF beams typically have a lower mean energy.^10^ This variation in energy spectrum will impact the behavior of the beam in media, and consequently calculated dose, for the CT‐number (Hounsfield unit) to relative electron density (RED) curve of a given treatment planning system (TPS).

The CT‐RED curve of the scanner on which the treatment planning CT is acquired has an impact on the calculated dose.^11^ In an effort to expedite palliative treatment, investigations into the use of previously acquired diagnostic CT scans for treatment planning have been conducted in phantoms, and retrospectively on 3D patient plans.^11^, ^12^, ^13^, ^14^, ^15^, ^16^, ^17^, ^18^, ^19^ Omitting the need for a dedicated simulation CT reduces the time required by the patient at the cancer center, reduces potential treatment delays, and requires fewer treatment planning resources. These studies showed negligible clinical impact of using diagnostic CT scans for palliative treatment planning in the phantom and 3D setting. To the best of the authors’ knowledge, no studies have investigated the impact of variable CT‐RED curves with FFF beams and conformal VMAT techniques together. Prior to the clinical implementation of conformal treatment planning with FFF beams on diagnostic CTs for palliative radiotherapy, the dosimetric impact of these deviations (for a given fluence map) from conventional planning approaches must be evaluated.

The purpose of this study was to evaluate the dosimetric impact of VMAT planning with 6 FFF and 10 FFF energy spectra using CT images acquired from different vendors, and between different CT x‐ray tube energies for a single vendor, compared to a 6 MV flat beam in the palliative setting. This study did not evaluate plan quality or speed of delivery.

## 2 METHODS

### 2.1 CT‐RED curve acquisition

The Gammex RMI Electron Density CT Phantom (Gammex, Middleton, WI, USA) was used to establish CT‐RED curves. The phantom is comprised of a solid water disk 33 cm in diameter and 5 cm thick, with a series of 2.8 cm diameter inserts of known RED, ranging from 0.28 (lung) to 1.69 (cortical bone). The phantom was aligned on the imaging couch so that the known density inserts were perpendicular to the imaging plane, and an axial scan was acquired. The onboard region of interest tool was used to measure the average CT number within each density insert on the axial slice mid‐phantom. A 1‐cm region of interest was used for each insert. Scans were acquired at 120 kVp for the local CT simulator (Philips Big Bore RT, Philips, Amsterdam, Netherlands), and two local diagnostic CT scanners from different vendors (Toshiba Acquilion One, Canon Medical Systems Corporation, Ōtawara, Tochigi, Japan, and GE HD Discovery, GE HealthCare, Chicago, IL, USA).

A similar setup, acquisition, and measurement technique were applied to evaluate the effect of x‐ray tube energy for a single scanner. Scan sets were acquired at 100 and 140 kVp in addition to the standard 120 kVp of the Philips CT simulator. CT‐RED mappings for the three separate CT scanners, and the two additional energies for the CT simulator were entered into the Monaco Treatment Planning System (Monaco 6.1.2.0, Elekta Solutions AB, Stockholm, Sweden).

### 2.2 Dosimetric comparison and statistical analysis

A representative sample of 30 palliative patient plans, prescribed 800 cGy in a single fraction, were chosen for dosimetric comparison of the different CT‐RED curves. Fifteen thoracic plans, including the thoracic and cervical spine, mediastinum, sternum, and shoulder targets, and fifteen pelvic plans, including the lumbar spine, ilium, and femur targets, were included. Planning target volume (PTV) margins were most commonly a 1 cm expansion from the clinical target volume (range = 0.5 to 1.35 cm), clipped to the patient external contour where appropriate. The selected plans had PTVs that did not extend beyond 15 cm edge‐to‐edge in any dimension to make the best use of the FFF beam's peaked central profile.

All plans were originally 6 MV flat VMAT delivery, optimized with Monaco's x‐ray voxel Monte Carlo (XVMC) dose calculation engine. The approved treatment plans were recalculated for each CT‐RED curve for 6 MV flat, 6 FFF, and 10 FFF photon beam energies. Plans were recalculated, not reoptimized, to isolate the dosimetric impact of the varying CT‐RED curves.

Dosimetric values for comparison between plans included the dose to 95% and to the hottest 2% of the PTV (D95% [*n* = 30 plans], and D2% [*n* = 30 plans], respectively). When contoured, the maximum point dose (D0.03 cc) to the spinal canal (*n* = 25 plans) and the esophagus (*n* = 12 plans), and the mean dose (*D*_mean_) to the kidneys (*n* = 12 plans) were recorded. For comparison between recalculated plans, dosimetric values were normalized such that the PTV D95% = 800 cGy (100% of the prescription dose) for the reference plan (Philips CT Simulator at 120 kVp) for each patient at each photon beam energy.

A one‐way repeated measures analysis of variance (ANOVA) was used to determine statistically significant differences (*p* ≤ 0.05) between doses for plans calculated with CT‐RED curves from different vendors (Toshiba, GE, Philips at 120 kVp), and separately, CT‐RED curves acquired at 100, 120, and 140 kVp (Philips CT simulator) for each beam energy. Descriptive statistics are also reported. All analyses were performed in GraphPad Prism (Prism 10.2.3, GraphPad Software Inc, San Diego, USA).

## 3 RESULTS

### 3.1 CT‐RED curve acquisition

The average CT numbers for the Gammex RMI Electron Density CT Phantom for the CT simulator and two diagnostic CT scanners, as well as the two additional x‐ray tube energies for the CT simulator are reported in Table 1. Air (RED = 0.001) was assigned a CT number of −1024 for each curve. Variations in CT number for the same material composition ranged from 10 (water) to 50 (cortical bone 50% CaCO_3_) between CT vendors for the same x‐ray tube energy (120 kVp), and from 4 (water) to 306 (cortical bone 50% CaCO_3_) for a difference in x‐ray tube energy of 40 kVp (100 vs. 140 kVp) on the same scanner.

**TABLE 1:** CT‐RED mappings of the Gammex RMI electron density phantom.

	CT number				
Gammex RMI insert RED, Material	Toshiba Acquilion One Diagnostic CT (120 kVp)	GE HD Discovery Diagnostic CT (120 kVp)	Philips Big Bore RT CT Simulator (100 kVp)	Philips Big Bore RT CT Simulator (120 kVp)	Philips Big Bore RT CT Simulator (140 kVp)
0.28, Lung (LN‐300; 0.30 g/cm^3^)	−683	−669	−696	−691	−691
0.40, Lung (LN‐450; 0.45 g/cm^3^)	−553	−531	−554	−553	−552
0.90, Adipose	−79	−83	−92	−84	−78
1.00, Water	0	−10	−2	−5	−6
1.09, Inner bone	234	221	260	222	199
1.28, Cortical bone (30% CaCO_3_)	466	443	506	447	413
1.69, Cortical bone (50% CaCO_3_)	1218	1168	1387	1192	1081

Abbreviation: CT, computed tomography.

### 3.2 Dosimetric comparison and statistical analysis

The PTV D95%, PTV D2%, spinal canal maximum point dose (D0.03 cc), esophagus maximum point dose (D0.03 cc), and mean dose for the combined kidneys structure were compared between plans calculated with different CT‐RED curves using a one‐way repeated measures ANOVA. A statistically significant difference (*p* ≤ 0.05) was found for 6 MV, 6 FFF, and 10 FFF beams between Toshiba, GE, and Philips scanners at 120 kVp for the PTV D95%, PTV D2%, and spinal canal D0.03 cc, and for the esophagus D0.03 cc with 6 MV flat, and mean kidneys dose for 10 FFF (Table 2).

**TABLE 2:** Results of one‐way repeated measures ANOVA comparing *n* plans calculated with CT‐RED curves acquired from diagnostic CT scanners (Toshiba and GE) and the radiotherapy CT simulator (Philips) for 6 MV, 6 FFF and 10 FFF. Statistical significance (bolded) is set at *p* ≤ 0.05.

		Mean ± SD (normalized to Philips CT Simulator at 120 kVp)			
		Toshiba (120 kVp)	GE (120 kVp)	Philips (120 kVp)	*p*‐value
PTV D95% (*n* = 30)	6 MV	1.001 ± 0.001	1.000 ± 0.001	**1.000** ± 0.000	**<0.001**
	6 FFF	1.001 ± 0.001	1.000 ± 0.001	**1.000** ± 0.000	**<0.001**
	10 FFF	1.001 ± 0.001	1.000 ± 0.001	**1.000 **± 0.000	**<0.001**
PTV D2% (*n* = 30)	6 MV	1.002 ± 0.010	1.000 ± 0.010	**1.000** ± 0.010	**<0.001**
	6 FFF	1.002 ± 0.085	1.000 ± 0.085	**1.000** ± 0.085	**<0.001**
	10 FFF	1.001 ± 0.088	0.999 ± 0.087	**1.000 **± 0.088	**<0.001**
Spinal canal D0.03 cc (*n* = 25)	6 MV	1.002 ± 0.352	0.999 ± 0.351	**1.000** ± 0.352	**0.009**
	6 FFF	1.002 ± 0.363	1.000 ± 0.362	**1.000** ± 0.362	**<0.001**
	10 FFF	1.003 ± 0.373	1.001 ± 0.372	**1.000 **± 0.372	**0.010**
Esophagus D0.03 cc (*n* = 12)	6 MV	1.003 ± 0.466	1.002 ± 0.465	**1.000** ± 0.465	**0.025**
	6 FFF	1.002 ± 0.479	0.999 ± 0.478	**1.000** ± 0.478	0.075
	10 FFF	1.001 ± 0.496	0.999 ± 0.495	**1.000 **± 0.494	0.670
Kidneys *D*_mean_ (*n* = 12)	6 MV	1.001 ± 0.722	1.000 ± 0.722	**1.000** ± 0.722	0.061
	6 FFF	1.001 ± 0.749	1.000 ± 0.748	**1.000** ± 0.748	0.052
	10 FFF	1.001 ± 0.756	1.000 ± 0.755	**1.000 **± 0.756	**0.023**

Abbreviations: CT, computed tomography; FFF, flattening filter free; PTV, planning target volume.

Comparing x‐ray tube energies for the CT simulator, a statistically significant difference was found for 6 MV flat, 6 FFF, and 10 FFF beams for the PTV D95% and PTV D2%; for the spinal canal D0.03 cc, esophagus D0.03 cc, and kidneys mean dose for the 6 FFF beam; and for the esophagus D0.03 cc for 6 MV flat (Table 3).

**TABLE 3:** Results of one‐way repeated measures ANOVA comparing *n* plans calculated with CT‐RED curves acquired at x‐ray tube energies of 100, 120, and 140 kVp for 6 MV, 6 FFF and 10 FFF. Statistical significance (bolded) is set at *p* ≤ 0.05.

		Mean ± SD (normalized to Philips CT Simulator at 120 kVp)			
		100 kVp (Philips)	120 kVp (Philips)	140 kVp (Philips)	*p*‐value
PTV D95% (*n* = 30)	6 MV	1.001 ± 0.001	**1.000** ± 0.000	0.999 ± 0.001	**<0.001**
	6 FFF	1.001 ± 0.001	**1.000** ± 0.000	1.000 ± 0.001	**<0.001**
	10 FFF	1.001 ± 0.001	**1.000 **± 0.000	1.000 ± 0.001	**0.001**
PTV D2% (*n* = 30)	6 MV	1.001 ± 0.009	**1.000** ± 0.010	1.000 ± 0.010	**<0.001**
	6 FFF	1.002 ± 0.085	**1.000** ± 0.085	0.999 ± 0.084	**<0.001**
	10 FFF	1.001 ± 0.088	**1.000 **± 0.088	0.999 ± 0.087	**<0.001**
Spinal canal D0.03 cc (*n* = 25)	6 MV	1.000 ± 0.352	**1.000** ± 0.352	0.999 ± 0.351	0.174
	6 FFF	1.001 ± 0.363	**1.000** ± 0.362	0.999 ± 0.362	**0.039**
	10 FFF	1.002 ± 0.372	**1.000 **± 0.372	1.001 ± 0.372	0.071
Esophagus D0.03 cc (*n* = 12)	6 MV	1.003 ± 0.466	**1.000** ± 0.465	0.998 ± 0.464	**0.008**
	6 FFF	1.001 ± 0.479	**1.000** ± 0.478	0.998 ± 0.477	**0.021**
	10 FFF	1.002 ± 0.495	**1.000 **± 0.494	1.001 ± 0.495	0.511
Kidneys *D*_mean_ (*n* = 12)	6 MV	1.000 ± 0.722	**1.000** ± 0.721	1.000 ± 0.721	0.277
	6 FFF	1.001 ± 0.748	**1.000** ± 0.747	1.000 ± 0.747	**0.034**
	10 FFF	1.001 ± 0.756	**1.000 **± 0.755	1.000 ± 0.756	0.528

Abbreviations: CT, computed tomography; FFF, flattening filter free; PTV, planning target volume.

**FIGURE 1:**
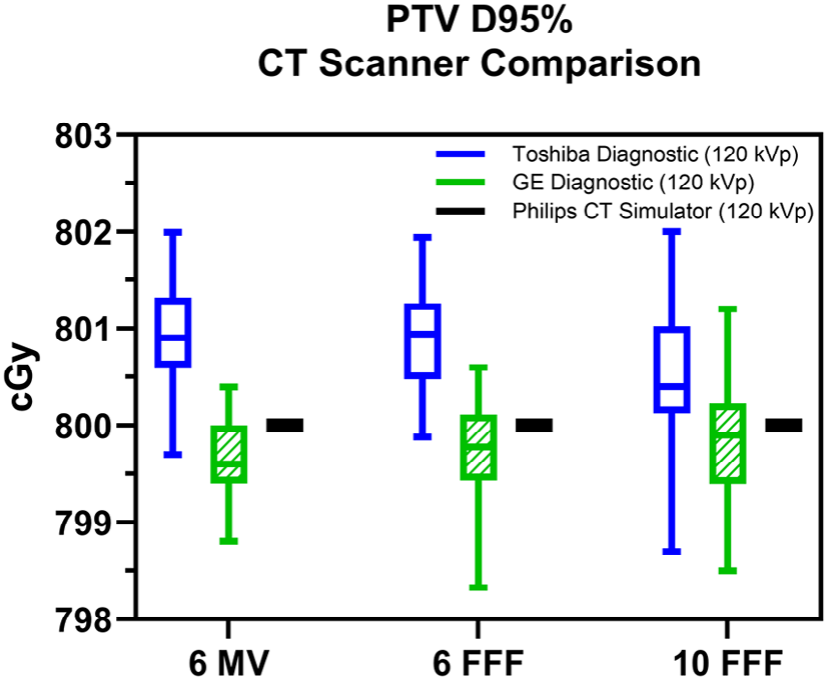
Variation of PTV D95% for different CT scanners. Box plots (median, interquartile range, maximum and minimum) comparing the PTV D95% calculated with CT‐RED curves acquired with the Toshiba Acquilion One (solid blue) and GE HD Discovery (hashed green) diagnostic scanners, compared to the Philips CT simulator (solid black reference) for 6 MV flat, 6 FFF, and 10 FFF beam energies. CT, computed tomography; CT‐RED, CT‐relative electron density; FFF, flattening filter free; PTV, planning target volume.

**FIGURE 2:**
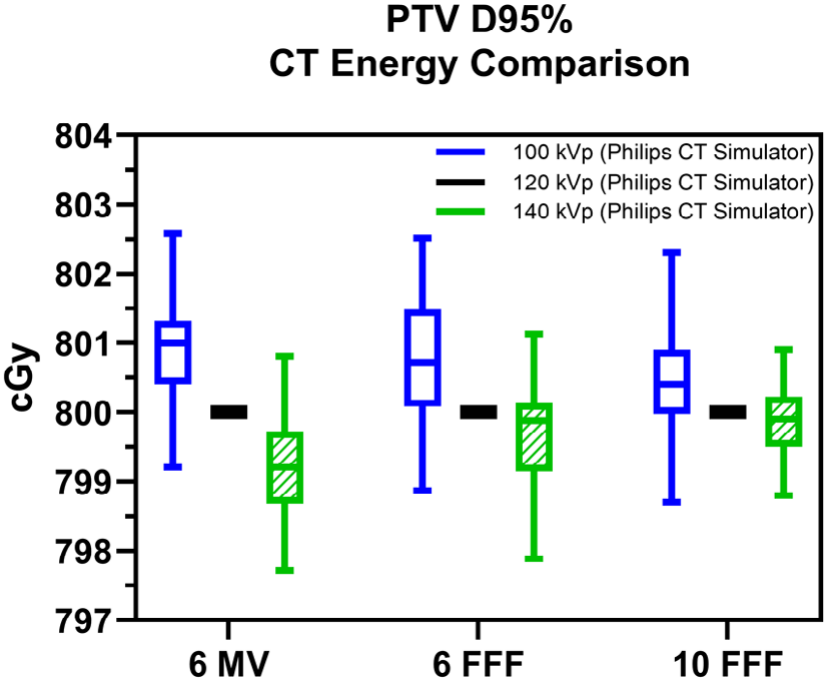
Variation of PTV D95% for different x‐ray tube energy. Box plots (median, interquartile range, maximum and minimum) comparing the PTV D95% calculated with CT‐RED curves acquired at 100 kVp (solid blue) and 140 kVp (hashed green) x‐ray tube energies, compared to the standard 120 kVp (solid black reference) of the Philips CT simulator for 6 MV flat, 6 FFF, and 10 FFF beam energies. CT, computed tomography; CT‐RED, CT‐relative electron density; FFF, flattening filter free; PTV, planning target volume.

The mean absolute magnitude of these dosimetric differences was at most 0.3% (less than 3 cGy in the case of an 800 cGy single fraction dose) of the dose measured with the Philips CT simulator CT‐RED curve at 120 kVp for each photon beam energy. Absolute values of the PTV D95% dosimetric comparison are shown in Figures 1 and 2.

## 4 DISCUSSION AND CONCLUSION

The purpose of this study was to assess dosimetric differences between palliative FFF VMAT plans calculated with CT‐RED curves from diagnostic CT scanners and a dedicated CT simulator, and to assess the impact of CT x‐ray tube energy on CT‐RED curves and subsequent dose calculations.

While the results show a statistically significant difference among multiple DVH metrics between both the comparison of diagnostic and simulation CT scanners, and between x‐ray tube energy, the differences are of negligible clinical impact, with a maximum mean difference from the reference plan set (Philips CT simulator at 120 kVp) of 0.3% for both flat and FFF beams. The statistical significance demonstrates that a small but consistent variation in the CT‐RED curve, as is the case with curves from different scanners or for different x‐ray tube energies, results in a predictable calculated dose difference. The greatest variation in CT number for a given material composition was for cortical bone (50% CaCO_3_), ranging from 50 between scanners, to upward of 300 for varying x‐ray tube energy. All the plans evaluated in this analysis included bony targets, highlighting that dose deposition in target structures with the greatest variation in CT number due to differences in scanner or energy is not appreciably different.

It is noted that FFF plans were recalculated, and not reoptimized, as we sought to investigate the impact of the different beam energy spectrum on dosimetry. In order to evaluate potential differences, the fluence maps between plans remained the same. As a result, the authors cannot comment on the difference in delivery time, conformity index, or heterogeneity index between appropriately optimized flat and FFF beams in this study, though it has been established previously that FFF's increased dose rate decreases treatment time, with acceptable conformity and heterogeneity when treating the same target volumes.^20^, ^21^

This study was limited by the institution's number of diagnostic CT vendors available for use (2), x‐ray tube energies available on the CT simulator (100, 120, and 140 kVp), and the number of available treatment planning beam energies for VMAT delivery (6 MV flat, and 6 and 10 FFF). It is also acknowledged that the 30 plans used for assessment were chosen to represent common palliative treatments and that special considerations, such as the presence of high‐density hardware, were not evaluated. Despite these limitations, this study reflects the negligible clinical impact of varying CT‐RED curves and photon beam energy in the setting of routine palliative radiotherapy.

In addition to potential differences in acquisition parameters between diagnostic and simulator CTs, it is important to address differences in patient positioning. The diagnostic CT is equipped with a curved couch‐top, dissimilar to the flat couch‐top used for radiotherapy. If diagnostic imaging is used for treatment planning and delivery, effort must be made to reproduce the diagnostic setup on the treatment couch. Previous studies have overcome this barrier by using full‐body vac‐bags curved to mimic the curvature of a diagnostic couch and other passive immobilization devices, as required.^12^, ^13^ Custom in‐house solutions such as a curved foam board may also be appropriate. The use of six‐degree‐of‐freedom treatment couches can also help with rotation that may be present in the diagnostic setup. Advances in autocontouring, autoplanning, and synthetic CT generation have introduced real‐time adaptive planning based on CBCT acquisition at the time of treatment, which is also a potential solution for addressing anatomic differences.^16^ A pilot patient study assessing the reproducibility of the diagnostic setup on radiotherapy couch may help inform appropriate PTV margins for the clinical implementation of conformal treatment of plans created on previously acquired imaging.

The results of this study confirm that diagnostic scans acquired on GE and Toshiba CT scanners, different from the Philips CT simulator associated with the TPS, are appropriate for VMAT treatment planning in the palliative setting with FFF photon beams.

## AUTHOR CONTRIBUTIONS

M.V. was responsible for data acquisition, analysis, interpretation, and manuscript preparation. L.B. was responsible for study design and data acquisition. E.A. was responsible for study design and data interpretation. All authors have critically revised and approved the manuscript for publication. All authors agree to be accountable for all aspects of the work.

## CONFLICT OF INTEREST STATEMENT

The authors declare no conflicts of interest.
